# TMAO Suppresses Megalin Expression and Albumin Uptake in Human Proximal Tubular Cells Via PI3K and ERK Signaling

**DOI:** 10.3390/ijms23168856

**Published:** 2022-08-09

**Authors:** Stefania Kapetanaki, Ashok Kumar Kumawat, Katarina Persson, Isak Demirel

**Affiliations:** 1 School of Medical Sciences, Örebro University, Campus USÖ, 701 82 Örebro, Sweden; 2 Nephrology Department, Karolinska University Hospital, 141 86 Huddinge, Sweden; 3 Cardiovascular Research Center, Faculty of Medicine and Health, Örebro University, 702 81 Örebro, Sweden; 4 iRiSC—Inflammatory Response and Infection Susceptibility Center, Faculty of Medicine and Health, Örebro University, 702 81 Örebro, Sweden

**Keywords:** TMAO, chronic kidney disease, megalin, albumin uptake, proximal tubular cells

## Abstract

Trimethylamine-N-oxide (TMAO) is a uremic toxin, which has been associated with chronic kidney disease (CKD). Renal tubular epithelial cells play a central role in the pathophysiology of CKD. Megalin is an albumin-binding surface receptor on tubular epithelial cells, which is indispensable for urine protein reabsorption. To date, no studies have investigated the effect of TMAO on megalin expression and the functional properties of human tubular epithelial cells. The aim of this study was first to identify the functional effect of TMAO on human renal proximal tubular cells and second, to unravel the effects of TMAO on megalin-cubilin receptor expression. We found through global gene expression analysis that TMAO was associated with kidney disease. The microarray analysis also showed that megalin expression was suppressed by TMAO, which was also validated at the gene and protein level. High glucose and TMAO was shown to downregulate megalin expression and albumin uptake similarly. We also found that TMAO suppressed megalin expression via PI3K and ERK signaling. Furthermore, we showed that candesartan, dapagliflozin and enalaprilat counteracted the suppressive effect of TMAO on megalin expression. Our results may further help us unravel the role of TMAO in CKD development and to identify new therapeutic targets to counteract TMAOs effects.

## 1. Introduction

Chronic kidney disease (CKD) negatively affects the quality of life of a great number of individuals. More specifically, the global prevalence of CKD was estimated to be 697.5 million in 2017, a number that represents 9.1% of the world’s population [1]. The same year, CKD resulted in 35.8 million disability-adjusted life years (DALYs) and the global mortality of CKD was 1.2 million [1]. Several studies have highlighted the substantial economic burden of CKD [2,3,4] on society. Trimethylamine-N-oxide (TMAO) is a uremic toxin found to be increased in the plasma of CKD patients compared to non-CKD individuals [5]. TMAO is the product of oxidation of trimethylamine (TMA) in the liver by flavin monooxygenase 3 (FMO3) and is physiologically eliminated in the urine [6]. TMA is generated from the metabolism of choline, L-carnitine and phosphatidylcholine by the gut microbiota [6]. Eggs, dairy products, red meat and fish are the main dietary sources of choline, phosphatidylcholine and L-carnitine [7]. TMA is transferred to the liver through circulation [6]. In CKD patients, alteration in the gut microbiota leads to gut mucosa inflammation and breakdown of enterocyte tight junctions [8]. Consequently, uremic toxins, including TMA, translocate from the gut to the bloodstream, leading to increased supply of TMA to the liver and, consequently, increased TMAO production [8]. Altered gut microbiome in combination with eventually decreased urine production in advanced stages of CKD, results in the aforementioned increase in plasma concentration of TMAO in CKD patients. TMAO levels in the serum were inversely correlated with glomerular filtration rate (GFR) [9]. The more advanced the stage of CKD, the lower the observed GFR and the higher the serum levels of TMAO [9]. Several clinical studies have tried to elucidate the role of TMAO in CKD [5,9,10,11,12,13,14]. High TMAO serum levels in CKD patients were associated with increased all-cause mortality and poorer 5-year survival compared to CKD patients with low TMAO and healthy individuals [5,9,10,11]. Moreover, increased TMAO concentration was associated with increased risk of cardiovascular events and greater atherosclerosis burden in CKD patients [12,13]. Finally, hemodialysis patients with higher plasma levels of TMAO had increased hospitalization rates compared to those with lower concentration of TMAO [14].

Apart from the glomerulus, which has been the subject of extensive research, the renal tubulointerstitium also contributes to the progression of CKD [15]. It consists of tubular cells, peritubular endothelium of the capillaries, extracellular matrix, pericytes, fibroblasts and immune cells [16]. The role of TMAO in the renal tubulointerstitium has also been a subject of investigation [5,17]. Increased TMAO in a CKD mice model was associated with tubulointerstitial fibrosis [5]. We have previously shown that TMAO induces proliferation of human renal fibroblasts and collagen production via the Akt/mTOR pathway [17]. Tubulointerstitial fibrosis and inflammation lead, in the context of CKD, to nephron loss and progressively declined GFR [15,18]. Furthermore, tubular epithelial cells play a central role in the initiation of tubulointerstitial inflammation and fibrosis, thereby the progression of CKD [15,18]. Proteinuria, a marker of CKD progression, is often a result of glomerular defects and leads to tubular cell activation [15,18]. More specifically, tubular cells exposed to a high-protein glomerular filtrate, start to de-differentiate [18]. Being in the latter state of the disease, these cells produce profibrotic and proinflammatory molecules that recruit fibroblasts and inflammatory cells [15].

Megalin and cubilin are two receptors forming a receptor complex on tubular cells and this receptor complex has been shown to be altered during CKD [19]. This receptor complex reabsorbs albumin and other proteins filtered in the proximal tubules by glomerulus [18]. Cubilin is the receptor required for the binding of albumin and megalin is necessary for the internalization of albumin into the tubular cell and initialization of cellular signaling [18,20,21,22]. Under the state of proteinuria, the capacity of cubilin and megalin is overwhelmed, leading to an increased protein delivery to the distal tubule [18]. Apart from that, there are studies supporting a reduction in the expression of megalin during CKD [19]. However, the mechanisms behind the reduced megalin expression are rather unclear.

To date, there are no studies that have investigated the effect of TMAO on the functional properties of tubular epithelial cells. Moreover, there is no data regarding the molecular mechanism through which this effect may be exerted. Such studies would help us to further clarify the role of TMAO in CKD and identify new therapeutic targets for this disease. The aim of this study was first to identify the functional effect of TMAO on human renal proximal tubular cells and second, to unravel the effects of TMAO on the megalin-cubilin receptor expression.

## 2. Results

### 2.1. Gene-Disease Associations in TMAO-Treated Human Renal Proximal Tubular Cells

**Figure 1:**
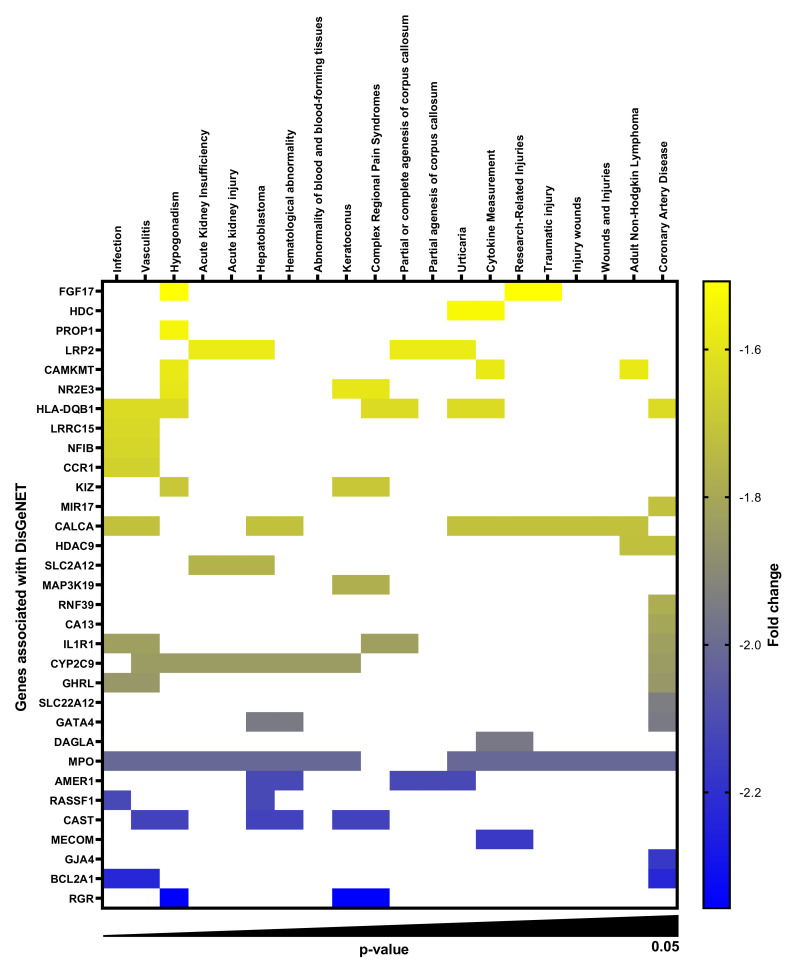
TMAO-downregulated genes in proximal tubular cells with DisGeNET association. Heat map showing the gene-disease association (*p* < 0.05) profile of 32 significantly downregulated genes (fold change ≥ 1.5) in proximal tubular cells after 6 h of TMAO 300 µM stimulation compared to unstimulated cells. Rows represent genes and columns represent DisGeNET diseases.

**Figure 2:**
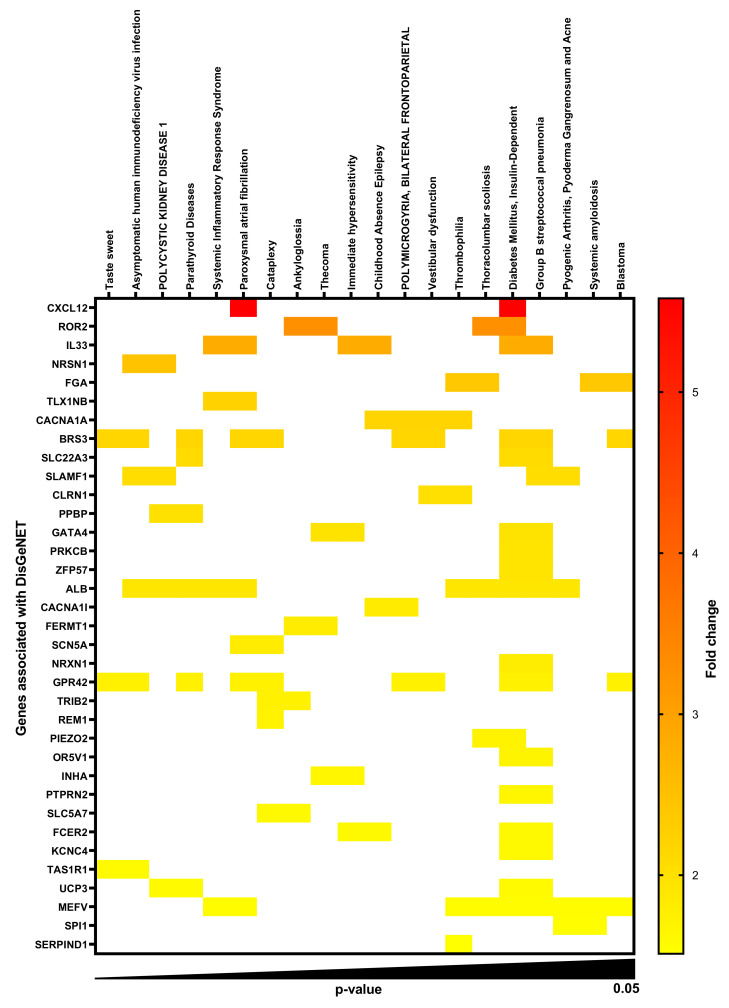
TMAO-upregulated genes in proximal tubular cells with DisGeNET association. Heat map showing the gene-disease association (*p* < 0.05) profile of 35 significantly upregulated genes (fold change ≥ 1.5) in proximal tubular cells after 6 h of TMAO 300 µM stimulation compared to unstimulated cells. Rows represent genes and columns represent DisGeNET diseases.

Total RNA was isolated from HK-2 human renal proximal tubular cells stimulated with TMAO, and a whole genome microarray analysis was conducted. Microarray enrichment analysis without multiple correction revealed 168 significantly (*p* < 0.05) upregulated (Supplementary Table S1) and 121 significantly downregulated (Supplementary Table S2) genes with a ≥1.5-fold change in comparison to unstimulated HK-2 cells after 6 h. DisgeNET disease enrichment analysis on the upregulated (Figure 1, Supplementary Table S3) and downregulated (Figure 2, Supplementary Table S4) genes revealed several gene-disease associations (GDAs, *p* < 0.05). We found that acute kidney insufficiency, acute kidney injury and polycystic kidney disease were highlighted as enriched in the DisgeNET analysis. The upregulated and downregulated genes found to have significant associations with various diseases are listed in Supplementary Tables S5 and S6. Furthermore, looking at the genes associated with polycystic kidney disease, acute kidney insufficiency and acute kidney injury, we found uncoupling protein 3 (UCP3), albumin (ALB), pro-platelet basic protein (PPBP), solute carrier family 22 member 12 (SLC22A12), cytochrome P450 family 2 subfamily C member 9 (CYP2C9), myeloperoxidase (MPO) and LDL receptor related protein 2 (LRP2) to be altered by TMAO. Of these genes, we found LRP2 to be of particular interest for further evaluation. LRP2 encodes for megalin, a transmembrane protein on the apical surface of proximal tubular cells involved in reabsorption of proteins.

### 2.2. TMAO Decreases Protein Expression of Megalin, but Not Cubilin, in Proximal Tubular Cells

**Figure 3:**
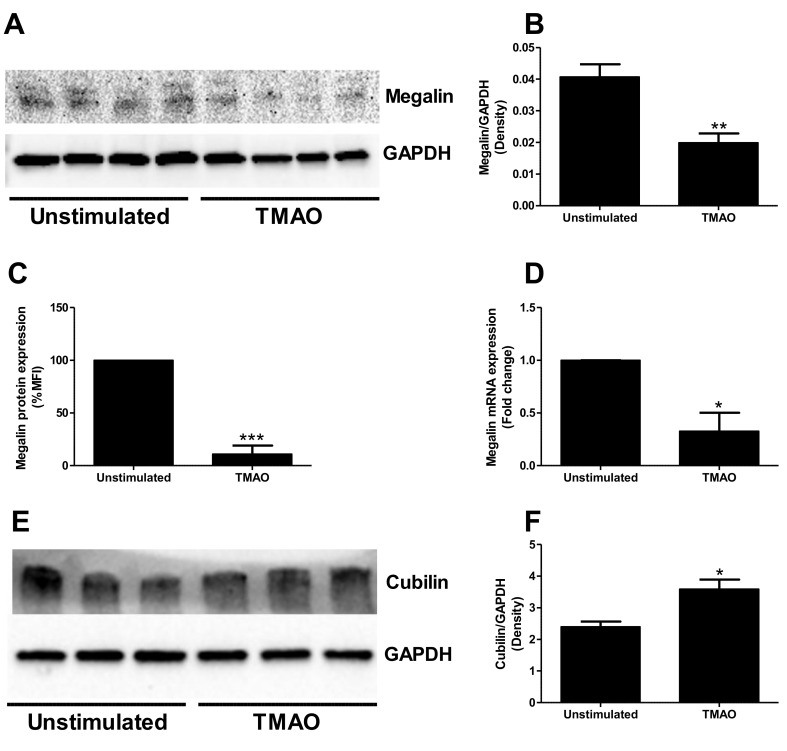
TMAO decreased megalin expression in proximal tubular cells. HK-2 cells were stimulated with TMAO (300 µM) for 24 h (**A**–**F**) followed by Western blot analysis (**A**,**B**,**E**,**F**), flow cytometry analysis (**C**) and RT-PCR to evaluate megalin (**A**–**D**) and cubilin (**E**,**F**) expression. GAPDH was used as a loading control. Data are presented as mean ± SEM (n = 3–4) independent experiments. MFI, mean fluorescence intensity. Asterisks denote statistical significance compared to unstimulated cells (* *p* < 0.05, ** *p* < 0.01, *** *p* < 0.001).

We then proceeded with investigating whether TMAO affects megalin expression at the protein level. TMAO stimulation of HK-2 cells for 24 h caused a decrease in protein expression of megalin compared to unstimulated cells (Figure 3A,B). These results were verified with flow cytometry (Figure 3C) and RT-PCR (Figure 3D). However, TMAO stimulated HK-2 cells exhibited significantly increased expression of cubilin, the protein that forms a receptor complex with megalin [18], compared to unstimulated cells (Figure 3E,F). Taken together, these results show that TMAO can downregulate megalin at the gene and protein level in HK-2 cells.

### 2.3. TMAO Reduces Albumin Uptake by Proximal Tubular Cells

**Figure 4:**
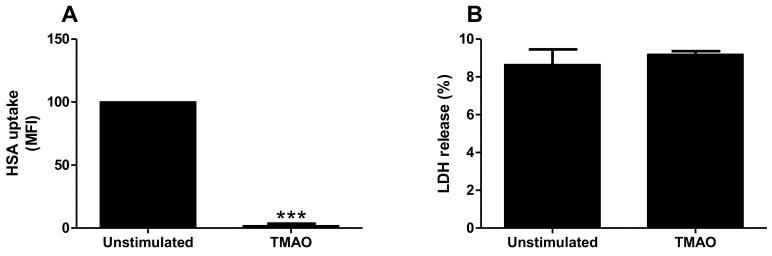
TMAO reduced albumin uptake in proximal tubular cells. HK-2 cells were stimulated with TMAO (300 µM) for 24 h (**A**,**B**) and HSA uptake (**A**) and LDH release (**B**) were evaluated. Data are presented as mean ± SEM (n = 3–6 independent experiments). HSA, human serum albumin. MFI, mean fluorescence intensity. Asterisks denote statistical significance compared to unstimulated cells (*** *p* < 0.001).

We continued to investigate if the decreased megalin expression mediated by TMAO had functional effects on albumin uptake. Indeed, TMAO stimulation for 24 h significantly reduced human serum albumin (HSA) uptake when compared to unstimulated cells (Figure 4A). This reduction was not a consequence of increased cell death, as verified with the LDH assay (Figure 4B). The intracellular uptake of HSA by HK-2 cells was also validated with confocal microscopy (data not shown). These results suggest that TMAO reduces albumin uptake by proximal tubular cells, possibly via the downregulation of megalin.

### 2.4. High Glucose Exposure Reduces Megalin Expression

**Figure 5:**
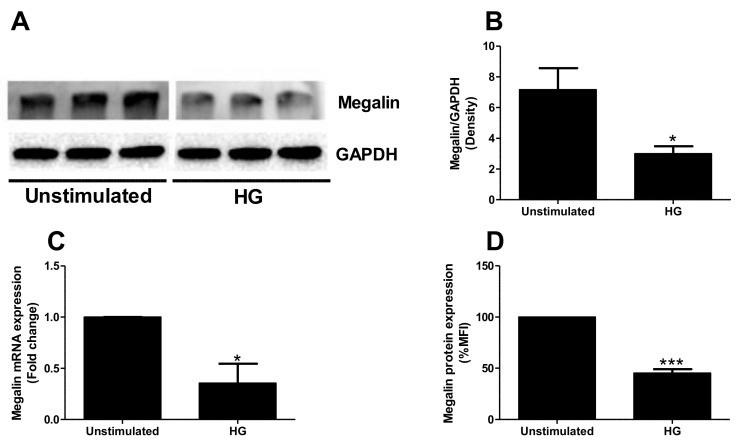

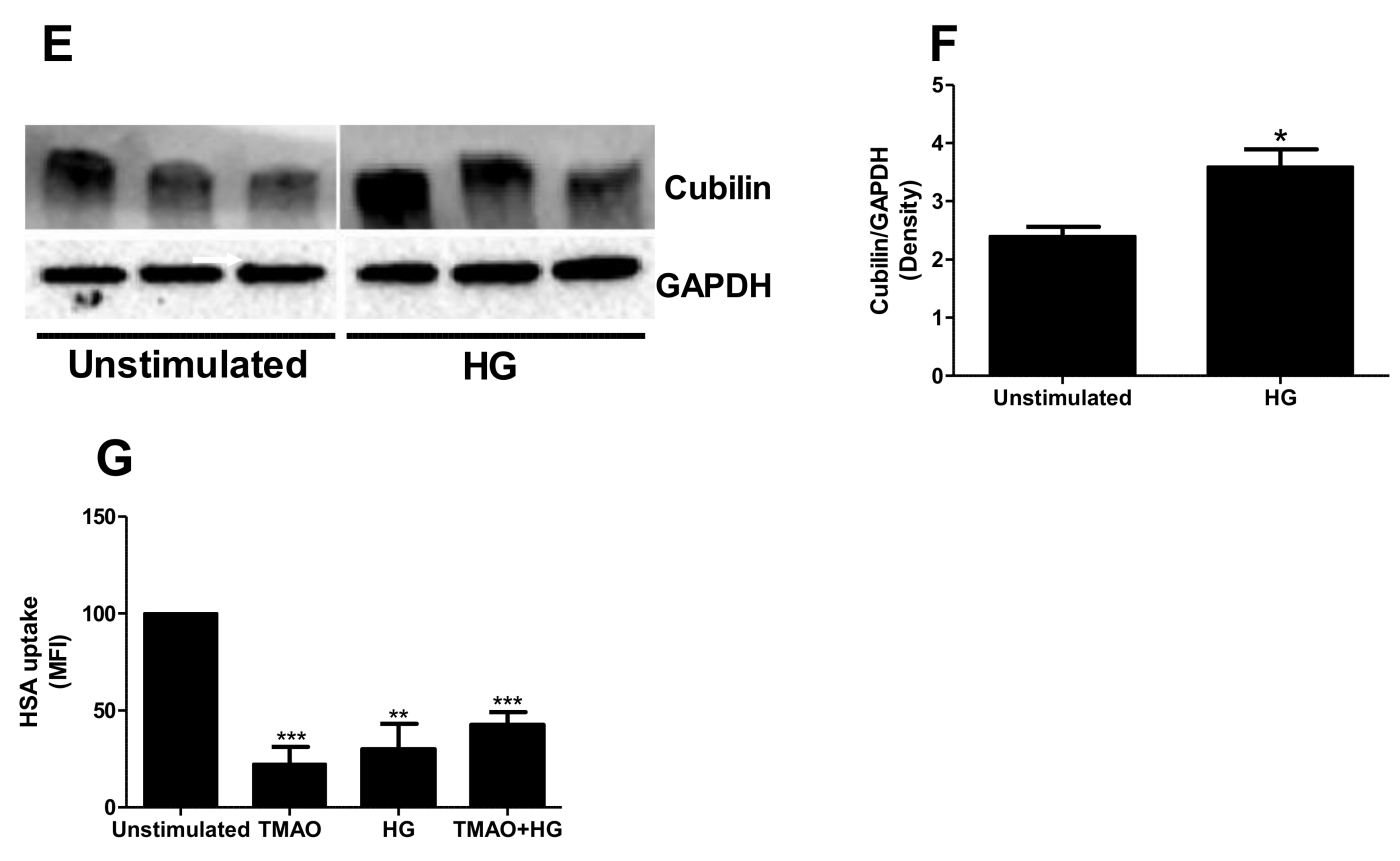
High glucose decreased megalin expression and albumin uptake in proximal tubular cells. HK-2 cells were stimulated with high glucose (30 mM) for 24 h (**A**–**F**) followed by Western blot analysis (**A**,**B**,**E**,**F**), flow cytometry analysis (**D**) and RT-PCR to evaluate megalin (**C**) and cubilin (**E**,**F**) expression. HSA uptake was also evaluated after 24 h of either high glucose (30 mM), TMAO (300 µM) or in combination, stimulation (**G**). GAPDH was used as a loading control. Data are presented as mean ± SEM (n = 3 independent experiments). HG, high glucose. Unstimulated (low glucose, 5 mM). MFI, mean fluorescence intensity. HSA, human serum albumin. Asterisks denote statistical significance compared to unstimulated cells (* *p* < 0.05, ** *p* < 0.01, *** *p* < 0.001).

High glucose is known for decreasing megalin expression and albumin uptake in proximal tubular cells [23]. Hence, we continued to investigate how high glucose and TMAO alone or in combination affects megalin expression and HSA uptake. In our study, high glucose treatment for 24 h decreased the protein expression of megalin (Figure 5A,B) compared to unstimulated cells. These results were verified with RT-PCR (Figure 5C) and flow cytometry (Figure 5D). High glucose, such as TMAO, caused an increase in cubilin protein expression (Figure 5E,F) in comparison to unstimulated cells. Moreover, in accordance with TMAO stimulation, high glucose exposure reduced HSA uptake by proximal tubular cells when compared to unstimulated cells (Figure 5G). Co-exposure of TMAO and high glucose also reduced HSA uptake compared to unstimulated cells (Figure 5G). However, this effect was not additive or synergistic. These results suggest that high glucose and TMAO affects proximal tubular cells similarly regarding megalin and cubilin expression and albumin uptake.

### 2.5. TMAO Suppresses Megalin Expression in Proximal Tubular Cells via PI3K and ERK

**Figure 6:**
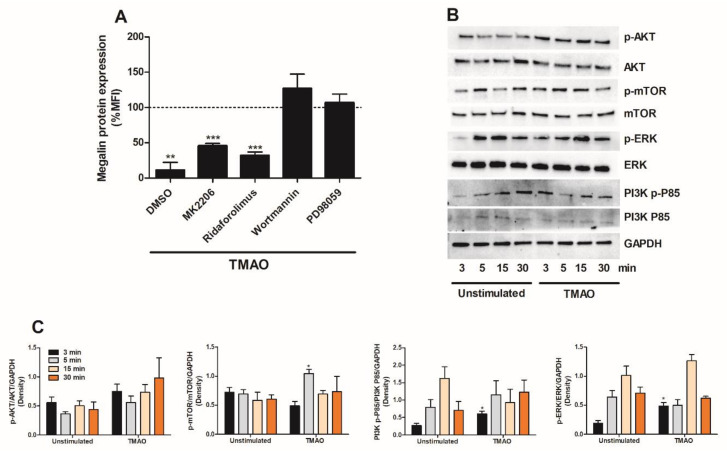
PI3K and ERK mediate TMAO-induced suppression of megalin expression. HK-2 cells were pre-incubated with DMSO (vehicle), Akt inhibitor MK-2206 (1 µM), mTOR inhibitor ridaforolimus (1 µM), PI3K inhibitor wortmannin (1 µM) or ERK inhibitor PD98059 (1 µM) for 1 h prior to TMAO stimulation (300 µM) for 24 h (**A**) followed by evaluating megalin protein expression with flow cytometry. Megalin protein expression is presented as % of vehicle control (DMSO or respective inhibitor alone), which is represented by the dotted line. Western blot analysis was conducted to identify differences in protein levels of p-Akt/Akt, p-mTOR/mTOR, p-ERK/ERK and PI3K p-P85/PI3K P85 after TMAO (300 µM) stimulation for 3, 5, 15 and 30 min (**B**,**C**). GAPDH was used as a loading control. Data are presented as mean ± SEM (n = 3 independent experiments). MFI, mean fluorescence intensity. Asterisks denote statistical significance compared to unstimulated cells (* *p* < 0.05, ** *p* < 0.01, *** *p* < 0.001).

The next step in our investigation was to identify which signaling molecules mediate the TMAO-induced suppression of megalin expression in proximal tubular cells. We found that inhibition of PI3K (wortmannin) and extracellular signal-regulated kinase (ERK) (PD98059) abolished TMAO’s ability to downregulate the expression of megalin after 24 h. However, inhibition of Akt (MK-2206) and mTOR (ridaforolimus) could not prevent TMAO from reducing the expression of megalin after 24 h (Figure 6A). Furthermore, Western blot results showed that HK-2 cells expressed significantly higher levels of p-mTOR, p-ERK and PI3K p-P85 after TMAO exposure compared to unstimulated cells (Figure 6B,C). In addition, we observed increased p-Akt, but this increase was not significant (Figure 6B,C). Taken together, our results showed that TMAO can activate the Akt, mTOR, PI3K and ERK signaling pathways, but only PI3K and ERK are indispensable in TMAO-induced decrease of megalin in HK-2 cells.

### 2.6. Candesartan, Dapagliflozin and Enalaprilat Counteract TMAOs Effect on Megalin

**Figure 7:**
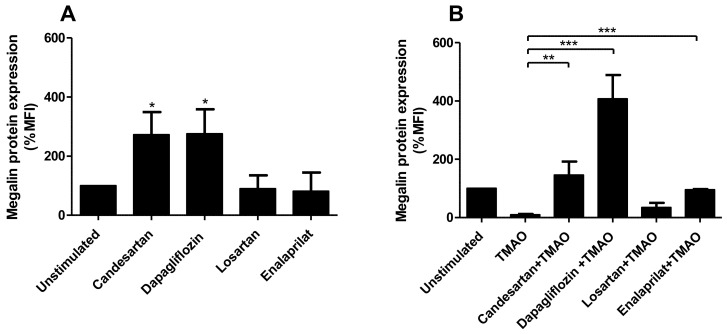
Candesartan, dapagliflozin and enalaprilat counteracts TMAO-induced reduction of megalin expression. HK-2 cells were treated with candesartan (10 µM), dapagliflozin (10 µM), losartan (10 µM) or enalaprilat (5 µM) for 12 h followed by assessment of megalin protein expression with flow cytometry analysis (**A**). Megalin expression was then evaluated in HK-2 cells stimulated with TMAO for 12 h, candesartan, dapagliflozin, losartan or enalaprilat was then added to the cells for an additional 12 h. Total exposure was 24 h for TMAO and 12 h for the drugs (**B**). Megalin protein expression is shown as % of unstimulated control. Data are presented as mean ± SEM (n = 3 independent experiments). MFI, mean fluorescence intensity. Asterisks denote statistical significance compared to unstimulated cells (* *p* < 0.05, ** *p* < 0.01, *** *p* < 0.001).

We then evaluated if candesartan (angiotensin II-receptor blocker), losartan (angiotensin II-receptor blocker), dapagliflozin (sodium/glucose cotransporter 2 (SGLT2) inhibitor) or enalaprilat (angiotensin-converting enzyme (ACE) inhibitor) could counteract the effects of TMAO on megalin expression. These drugs are currently extensively used against proteinuria at the clinical level. First, we evaluated how these drugs affect the basal expression of megalin in HK-2 cells. We found that candesartan and dapagliflozin, but not losartan or enalaprilat, increased the protein expression of megalin after 12 h compared to unstimulated cells (Figure 7A). We then moved on to stimulate HK-2 cells with TMAO for 12 h, and the respective drug was then added to the cells for an additional 12 h. Total exposure was 24 h for TMAO and 12 h for the drugs. We found that HK-2 cells treated with candesartan, dapagliflozin or enalaprilat, but not losartan, in combination with TMAO increased the expression of megalin in comparison to cells treated with only TMAO (Figure 7B). These results suggest that candesartan, dapagliflozin and enalaprilat counteract the suppressive effect of TMAO on megalin expression in proximal tubular cells.

## 3. Discussion

Several studies have investigated the role of TMAO in chronic kidney disease [5,9,10,11,12,13,14]. However, there are limited studies that have elucidated the effects of TMAO on human proximal tubular epithelial cell function [24,25,26]. Tubular epithelial cells contribute significantly to the initiation of tubulointerstitial inflammation and fibrosis, thereby to the development of CKD [15,18]. Understanding the interaction between TMAO and proximal tubular epithelial cells may help us identify new therapeutic targets for CKD. Our aim was to investigate the functional effect of TMAO on human renal proximal tubular cells and second, to unravel the effects of TMAO on the megalin-cubilin receptor expression.

We started by evaluating the global gene expression of HK-2 cells stimulated with TMAO. We found, using DisgeNET disease enrichment analysis, that genes altered by TMAO in HK-2 cells were associated with acute kidney insufficiency, acute kidney injury and polycystic kidney disease. Our findings are in agreement with previous studies showing a link between TMAO and kidney disease [5,9,10,11,12]. Evaluating the genes associated with kidney disease in the enrichment analysis, LRP2, which was downregulated, was found to be of particular interest. LRP2 encodes for megalin, a receptor that constitutes the major pathway for tubular reabsorption of filtered plasma proteins [19]. Hence, we continued to evaluate the effects of TMAO on megalin expression and function. We found that TMAO reduced the expression of megalin, but upregulated the co-receptor cubilin in HK-2 cells. Others have also found that megalin expression is reduced during CKD [19]. Megalin, cubilin and amnionless form a receptor complex in the apical membrane of proximal tubular cells and they are responsible for the reabsorption of filtered proteins, including albumin [19,27,28,29]. Megalin and amnionless are transmembrane proteins, whereas cubilin is a membrane protein [19,27,28]. The filtered albumin can bind to cubilin only or to both cubilin and megalin [19,27]. Megalin is necessary for the internalization of albumin but also for the internalization and recycling of cubilin [29,30,31]. Moreover, amnionless participates in the recycling of cubilin [19,27,29]. Based on this knowledge, there are two possible scenarios, which may explain the opposite effect of TMAO on megalin and cubilin expression in proximal tubular cells. The first, cubilin increase is a compensatory mechanism for the decrease of megalin. Second, reduction of megalin leads to reduced internalization of cubilin. Consequently, leading to reduced cubilin recycling and lysosomal degradation [28], which increases membrane accumulation of cubilin. Evaluating the functional effect of the altered megalin and cubilin expression, we found that albumin uptake was reduced by TMAO. Reduced albumin uptake by proximal tubular epithelial cells has been linked to CKD [32]. Taken together, our findings indicate that TMAO reduces megalin expression, which leads to reduced uptake of albumin by proximal tubular epithelial cells.

It is interesting that the effect of TMAO on megalin and cubilin expression and albumin uptake by proximal tubular cells was similar to that of high glucose exposure. Our findings are in accordance with the in vitro study by Peruchetti et al. [23]. An earlier in vivo study showed that rats with diabetes and diabetic kidney disease (DKD) fed with a high-fat diet, had increased urine protein and urine albumin levels compared to control rats [33]. Rats with DKD fed with the same diet, but with TMAO supplementation in their drinking water, exhibited higher urine protein and urine albumin levels compared to DKD rats with only a high-fat diet [33]. These findings suggest that high plasma TMAO levels exacerbate high glucose-caused proteinuria in diabetic animals. In our study, no difference in albumin uptake was identified between cells exposed to high glucose or high glucose in combination with TMAO. Hence, further studies are needed to investigate whether TMAO exacerbates high glucose/diabetes-caused proteinuria in humans.

Next, we proceeded by evaluating which signaling molecules mediate the TMAO-induced suppression of megalin expression. We found that TMAO suppresses megalin expression via PI3K and ERK, but not Akt and mTOR signaling in proximal tubular cells. However, TMAO had the ability to activate PI3K, ERK, Akt and mTOR signaling pathways in proximal tubular cells. We have previously shown that TMAO induced increased renal fibroblast proliferation and collagen production via Akt and mTOR, but not via PI3K [17]. TMAO has also been shown to promote vascular inflammation via the activation of ERK [34]. Furthermore, the PI3K/Akt/mTOR pathway regulates a variety of biological functions at the physiological and pathological levels. This signaling pathway is involved in cell proliferation, cell fate determination [35,36,37,38,39], cell viability and autophagy [40] at the physiological level. At the pathological level, the pathway has been associated with tumor growth, tumor metabolism and kidney disease [41,42,43,44]. Taken together, our findings indicate that PI3K and ERK, but not Akt and mTOR, mediate the effect of TMAO on megalin expression in proximal tubular cells.

We also investigated if candesartan (angiotensin II-receptor blocker), losartan (angiotensin II-receptor blocker), dapagliflozin (sodium/glucose cotransporter 2 (SGLT2) inhibitor) or enalaprilat (angiotensin-converting enzyme (ACE) inhibitor) could counteract the effects of TMAO on megalin expression. These drugs are currently extensively used against proteinuria in patients [45]. We found that candesartan, dapagliflozin and enalaprilat, but not losartan, counteracted the suppressive effect of TMAO on megalin expression. In addition, candesartan and dapagliflozin increased the basal expression of megalin in HK-2 cells. This is possibly one of the mechanisms through which candesartan and dapagliflozin reduces proteinuria in vivo. Our findings are in accordance with the findings of Hosojima et al. who demonstrated that angiotensin 1 (AT1) receptor blockade reversed the decrease in megalin expression mediated by angiotensin II in proximal tubular cells [46]. However, losartan, although an angiotensin receptor blocker, caused no change in megalin expression in proximal tubular cells in vitro. However, according to a previous study, enalapril and losartan reduced proteinuria in hypertensive rats by increasing the expression of cubilin [47]. Both drugs were shown to decrease megalin expression in these rats [47]. Although candesartan and losartan belong to the same group of drugs, they differed regarding their capacity to counteract the effect of TMAO on megalin. This could be related to their pharmacological differences. Candesartan binds with higher affinity to the AT_1_ receptor and this binding lasts longer compared to losartan [48,49,50,51,52], explaining the reduced anti-proteinuric action of losartan compared to candesartan [53]. Furthermore, in accordance with our study, Peruchetti et al. showed that the SGLT2 inhibitor phlorizin could counteract high glucose-induced downregulation of megalin in proximal tubular cells [23]. Taken together, our data suggests that candesartan, dapagliflozin and enalaprilat counteract the suppressive effect of TMAO, suggesting one mechanism behind their beneficial effects during proteinuria.

Some limitations of the present study are that in vitro experiments cannot completely mimic the complexity of the cell–cell interactions that exist in the tubulointerstitial renal tissue. Such interactions could possibly modify the effect of TMAO on megalin expression and tubular albumin uptake. In addition, we did not include the effect of TMAO on amnionless in our study, which, as mentioned above, forms a complex with megalin and cubilin for protein uptake.

In conclusion, we showed that TMAO affects proximal tubular cells by decreasing the protein expression of megalin. As a result, the albumin uptake capacity of proximal tubular cells was reduced in the presence of TMAO. The TMAO-induced decrease of megalin expression was mediated by PI3K and ERK and was reversed by candesartan, dapagliflozin and enalaprilat. These findings can be the basis of further research to investigate the contribution of TMAO to CKD development and to identify new therapeutic targets to counteract the effect of TMAO.

## 4. Materials and Methods

### 4.1. Cell Culture

The human immortalized proximal tubule epithelial cell line HK-2 (American Type Culture Collection (ATCC), Manassas, VA, USA) was used in this study. The HK-2 cell line was cultured in Dulbecco’s modified Eagle medium (DMEM) supplemented with 10% fetal bovine serum (FBS), 2 mM L-glutamine and 1 mM non-essential amino acids (all from Thermo Fisher Scientific, Waltham, MA, USA) at 37 °C in 5% CO_2_ atmosphere. Prior to experiments, the cells were serum starved overnight in DMEM supplemented with 2 mM L-glutamine and 1 mM non-essential amino acids. During the experiments, the cell culture medium was replaced with DMEM supplemented with 2 mM L-glutamine and 1 mM non-essential amino acids.

### 4.2. Stimulation of Renal Proximal Tubule Epithelial Cells

HK-2 cells were stimulated with TMAO (300 µM, Sigma-Aldrich, St. Louis, MO, USA), low glucose (5 mM, unstimulated) or high glucose (30 mM, Sigma-Aldrich) for 3 min to 24 h, depending on the experimental setup, at 37 °C in 5% CO_2_. The HK-2 cells were also pre-incubated with DMSO (vehicle), Akt inhibitor MK-2206 (1 µM, Selleckchem, TX, USA), mTOR inhibitor ridaforolimus (1 µM, Selleckchem), PI3K inhibitor wortmannin (1 µM, Selleckchem) and ERK inhibitor PD98059 (10 µM, Santa Cruz Biotechnology Inc., Heidelberg, Germany) for 1 h prior to TMAO stimulation. Supernatants, cell lysates and total ribonucleic acid (RNA) were collected and kept at –80 °C until further analysis.

### 4.3. Western Blot Analysis

HK-2 were lysed in radioimmunoprecipitation assay (RIPA) buffer supplemented with a protease and phosphatase inhibitor cocktail (Thermo Fisher Scientific). The protein concentrations of the samples were assessed using the DC protein assay (Bio-Rad Laboratories, Hercules, CA, USA). Protein samples and Laemmli buffer were mixed and boiled at 95 °C for 5 min. The samples (10–20 µg of protein) were separated with 4–15% SDS-polyacrylamide gel electrophoresis and then transferred to a polyvinylidene fluoride (PVDF) membrane (Bio-Rad Laboratories). Furthermore, 3% BSA was used to block the PVDF membrane for 1 h at room temperature. Megalin was detected using a mouse monoclonal antibody (sc-515772, Santa Cruz Biotechnology). Cubilin was detected using a mouse monoclonal antibody (sc-518089, Santa Cruz Biotechnology). Phospho-Akt (p-Akt) was detected using a rabbit monoclonal antibody (#4060, Cell signaling Technologies, Danvers, MA, USA). Phospho-mTOR (#5536, p-mTOR) was detected using a rabbit monoclonal antibody (Cell signaling Technologies). Phospho-P85 (p-P85, PI3K) was detected using a rabbit monoclonal antibody (#4228, Cell signaling Technologies). Phospho-ERK (p-ERK) was detected using a mouse monoclonal antibody (#9106, Cell signaling Technologies). Total Akt was detected using a mouse monoclonal antibody (#2920, Cell signaling Technologies). Total mTOR was detected using a rabbit monoclonal antibody (#2983, Cell signaling Technologies). Total P85 (PI3K) was detected using a rabbit monoclonal antibody (#4257, Cell signaling Technologies). Total ERK was detected using a rabbit monoclonal antibody (#4695, Cell signaling Technologies). GAPDH was used as loading control and was detected with a rabbit polyclonal antibody (sc-25778, Santa Cruz Biotechnology). All the primary antibodies were incubated overnight at 4 °C. The secondary antibodies, goat anti mouse IgG (horseradish peroxidase, HRP) (Santa Cruz Biotechnology) and goat anti rabbit IgG (HRP) (Santa Cruz Biotechnology) were used and incubated for 1 h at room temperature. Luminata Forte Western HRP Substrate (Merck Millipore) was used for developing the blots, as previously described [54].

### 4.4. RNA Isolation and Real Time RT-PCR

Total RNA was isolated from HK-2 cells using the E.Z.N.A. Total RNA Kit I (Omega, Bio-tek, GA, USA) following the manufacturer’s instructions. Quantification of the RNA was done using the Nano-Drop ND-1000 spectrophotometer (Wilmington, NC, USA). Furthermore, 100 ng total RNA was used for cDNA synthesis (20 µL reactions) using the High-capacity cDNA RT kit according to the kit’s instructions (Thermo Fisher Scientific). The real time RT-PCR was performed using Maxima SYBR Green qPCR Master Mix (Thermo Fisher Scientific), 5 ng cDNA and 250 nM of each megalin primer (96% amplification efficiency, forward: 5′-GTCTAACCGCACTGTGATAGCC-3′, reverse: 5′-CGGAAGTTTCCTCCCAATGTGG-3′), and glyceraldehyde 3-phosphate dehydrogenase (GAPDH) (94% amplification efficiency, forward: 5′-GTCTCCTCTGACTTCAACAGCG-3, reverse: 5′-ACCACCC TGTTGCTGTAGCCAA-3′). The primers were designed by Origene (Rockville, MD, USA) and synthesized by Eurofins MWG Synthesis GmbH (Munich, Germany). The PCR amplification was done using the CFX96 Touch Real-Time PCR Detection System (Bio-Rad Laboratories) according to following: desaturation at 95 °C for 10 min, 40 cycles of denaturation at 95 °C for 15 s and finally, annealing/extension at 60 °C for 60 s. The gene expression was assessed by the comparative Ct (ΔΔCt) method followed by normalization to the endogenous control GAPDH. GAPDH was validated in the experimental setup to be a suitable control. Fold difference was calculated as 2^−ΔΔCt^, as previously described [55]. A no-reverse transcriptase control and a no-amplification control were used as negative controls during the PCR.

### 4.5. Microarray Analysis

HK-2 cells were stimulated with TMAO (300 µM) for 6 h at 37 °C in 5% CO_2_. Total RNA was isolated from the HK-2 cells using the E.Z.N.A. Total RNA Kit I. RNA quality and integrity were evaluated using Agilent TapeStation 2200 platform (Agilent Technologies, Palo Alto, CA, USA) according to manufacturer instructions. The RNA integrity number (RIN) was 10 for all RNA samples. The Low Input Quick Amp WT Labelling Kit (Agilent) was used to prepare labelled cRNA according to manufacturer instructions. Hybridization of the labelled cRNA samples were done in a G2545A hybridization oven (Agilent) onto Agilent SurePrint G3 (v3) Human Gene Expression 8 × 60 k (Agilent Technologies) glass arrays according to manufacturer instructions and subsequently scanned with a G2505C array laser scanner (Agilent Technologies). Feature Extraction Software (version 10.7.3.1, Agilent Technologies) was used for image analysis and data extraction, as previously described [54]. Gene expression data are available in the GEO database with the accession number GSE210692.

### 4.6. Albumin Uptake and Cell Viability Assay

HK-2 cells were stimulated with TMAO (300 µM), low glucose (5 mM, unstimulated) or high glucose (30 mM) for 24 h at 37 °C in 5% CO_2_. After 24 h, 100 µg/mL human serum albumin-FITC (HSA-FITC, Jackson ImmunoResearch Europe Ltd., Cambridgeshire, UK) was added to the cells for 1 h at 37 °C. The cells were then washed with PBS 10 times and lysed with 0.1% SDS (in Milli-Q water). The lysate was transferred to a black 96-well plate and measured at *495* nm/519 nm using the Cytation 3 plate reader (BioTek, Winooski, VT, USA).

Cell viability was assessed by Pierce lactate dehydrogenase (LDH) cytotoxicity assay (Thermo fisher Scientific) following the manufacturer’s instructions [55].

### 4.7. Flow Cytometry

HK-2 cells were stimulated with TMAO (300 µM), low glucose (5 mM, unstimulated) or high glucose (30 mM) for 24 h at 37 °C in 5% CO_2_. The HK-2 cells were also treated with TMAO (300 µM) for 12 h followed by treatment with DMSO (vehicle), candesartan (10 µM, Santa Cruz Biotechnology), dapagliflozin (10 µM, Santa Cruz Biotechnology), losartan (10 µM, Santa Cruz Biotechnology) and enalaprilat (5 µM, Santa Cruz Biotechnology Inc) for an additional 12 h in the presence of TMAO. After 24 h, the cells were detached with Accutase (Sigma-Aldrich) and megalin was detected using the monoclonal mouse anti-human megalin/LRP2 Alexa Fluor 488-conjugated antibody (R&D Systems, Minneapolis, USA). Cells were stained with 0.25 µg antibody for 20 min at room temperature. The expression was evaluated using the Gallios (Beckman Coulter, Brea, CA, USA) flow cytometer with 488 nm laser and FL1 525/40 nm band-pass filter. The data were analyzed with Kaluza Flow Cytometry Analysis v1.3 (Beckman Coulter).

### 4.8. Data Analysis

All data shown are expressed as mean ± SEM. The differences between the groups were analyzed by unpaired Student’s *t*-test. Statistical significance of the differences was considered at *p* < 0.05. Microarray analysis was performed using Gene Spring GX version 14.9 (Agilent Technologies) after per chip and gene 75th percentile shift normalization of samples. Significantly expressed genes between groups was analyzed with Moderated T-Test (*p* < 0.05) with a fold change set at ≥1.5. Disease ontology enrichment was done with DisGeNET (v7.0) and the significance was set at a *p*-value < 0.05.

## Data Availability

Microarray data is uploaded to GEO database, GSE210692.

## Supplementary Materials

The following supporting information can be downloaded at: https://www.mdpi.com/article/10.3390/ijms23168856/s1.
